# Responding to the mental health needs of trafficked women

**DOI:** 10.1192/j.eurpsy.2021.55

**Published:** 2021-08-13

**Authors:** S. Oram

**Affiliations:** Health Service And Population Research; Section For Womens Mental Health, Institute of Psychiatry, Psychology and Neuroscience, London, United Kingdom

**Keywords:** Human trafficking, advocacy, Theory of change

## Abstract

**Background:**

Studies suggest a high prevalence of depression and PTSD among survivors of human trafficking in contact with shelter services. However, evidence for interventions to support the recovery of survivors of trafficking is lacking. The broader literature on PTSD and depression indicates that ongoing social stressors can exacerbate and perpetuate symptoms. Advocacy-based, or “casework”, interventions, which address current stressors and social support, may represent a promising avenue of enquiry. Objectives (1) Describe risk and protective factors for mental distress among trafficked people; (2) Present a preliminary theory of change describing how advocacy-based interventions may contribute to an improvement in mental health and wellbeing among survivors of human trafficking.

**Methods:**

(1) Survey of adult male and female survivors of trafficking in contact with shelter services in England; symptoms of depression, anxiety, and post-traumatic stress disorder were measured using the PHQ-9, GAD-7, and PCL-C. (2) Theory of change workshop and review of intervention studies that assessed the effectiveness of casework, client support, or advocacy interventions delivered in health or community settings to survivors of trafficking or vulnerable migrants.

**Results:**

150 survivors of trafficking participated in the survey, 98 women and 52 men. In multivariate analyses, psychological distress was associated with higher number of unmet needs and lacking a confidante, suggesting that practical and social support is important in facilitating mental health recovery. The theory of change identifies common components in advocacy interventions delivered to survivors of trafficking, and proposes pathways by which these components contribute to improved mental health.

**Disclosure:**

No significant relationships.

